# Ground Wheat Grain for Midlactation Cows: Challenging a Common Wisdom

**DOI:** 10.1100/2012/247941

**Published:** 2012-04-30

**Authors:** A. Nikkhah, F. Amiri, H. Amanloo

**Affiliations:** Department of Animal Sciences, Faculty of Agricultural Sciences, University of Zanjan, Zanjan 313-45195, Iran

## Abstract

The objective was to determine the effects of ground wheat grain (GW) inclusion rate, grinding extent (GE), and their interaction on lactating cow performance. Eight midlactation cows in 3 × 4 m individual boxes were used in a 4 × 4 replicated Latin square design study with 4 21 d periods. GW was fed at either 10% or 20% of diet dry matter (DM), as either finer or coarser particles. DM intake increased and net energy for lactation (NE_L_) intake tended to increase when GW was fed at 10% instead of 20% of diet DM. Milk energy yield, milk solids content and yield, and urine pH were unaffected. Fecal pH tended to increase at 20% versus 10% GW. Total tract apparent NDF, but not DM, digestibility tended to be greater for coarsely than finely GW and tended to be greater at 10% versus 20% GW. GW at 10% versus 20% of diet DM decreased blood BHBA and increased blood concentrations of total proteins and albumin. Data provide novel evidence that both finely and coarsely ground WG can be safely fed up to 20% midlactation cows. Commercial accessibility and cost will determine feeding preference of wheat grain to dairy cows.

## 1. Introduction

Wheat grain (WG: *Triticum spp.*) is an invaluable energy and amino acids source for livestock. Due to its usually fast and extensive rumen degradation, popular consumption by humans, and climatic, agronomical and economical constraints, WG has less commonly been fed to ruminants compared to corn and barley grains. Subsequent to oats, ground WG (GW) possesses the highest starch and protein degradation extent and rate among cereals [1, 2]. If not managed properly, increased dietary GW levels are, thus, more likely to cause subacute rumen acidosis (SARA), milk fat depression, and laminitis, when compared to corn and barley grains [3, 4]. However, such a SARA risk would exist rather shortly after feeding (44% versus 60% of total starch degraded after 4-h for barley and wheat grains, resp; [5]) because wheat and barley grains have almost similar degradation patterns after few hours after feeding [1, 6]. The traditional belief that feeding GW threatens rumen and ruminant health and production is prone to challenges. As such, in vitro and in vivo digestion and metabolic properties of feeding wheat cultivars to ruminants continue to receive research interests [7–9].

As WG becomes more accessible and cheaper, its dietary inclusion for lactating cows may be more feasible. However, such reports for feeding GW of different particle sizes are lacking. For adequate ruminal and small intestinal utilization, WG should be physically processed. Processing can improve DM digestion of WG in the whole gastrointestinal tract from 16–32% to 87–100% [14, 15]. Crushing, grinding and rolling are the common processing methods. Steam-processing may reduce ruminal degradation rate of rapidly fermentable grains (e.g., WG) via increased particle size, and likely by developing starch-protein and starch-lipids bonds in the endosperm [16]. However, steam-rolling equipment is very expensive to be established and be maintaind, particularly for medium and small size farms. Thus, in vivo data are required to determine if and to what extent differently ground WG, produced by hammer mills, can be fed to lactating cows. Uncovering optimum dietary inclusion rates of differently ground WG without other major investments in chemical and physical processing is of practical importance. Ground GW fed prepartum has recently been shown to increase blood glucose and calcium and improve milk production of periparturient cows and heifers [8, 17]. Nevertheless, GW inclusion rate in lactation diets in relation to processing methods and particle size has not been studied. Doepel et al. [7] found that steam-rolled WG may replace steam-rolled barley grain at 20% of diet DM for second-calf midlactation cows without compromising rumen conditions and nutrient digestibility. Feeding 20% of steam-rolled WG has been found feasible without any sodium bicarbonate in midlactation diets [18]. Whilst grinding is an easy-to-access and widely accessible technique to process WG, in vivo data are lacking on feeding lactating cows different levels of differently ground WG. In light of the higher rumen degradation rate of starch and protein in wheat grain than in barley grain, feeding GW at controlled levels was hypothesized to maintain feed intake and milk production. The primary objective was to determine effects of feeding a higher (20%) versus a lower (10%) level of coarsely versus finely GW in chopped alfalfa- hay- and wheat- straw- based mixed rations on feed intake, peripheral blood metabolites, and milk production of midlactation Holstein cows.

## 2. Materials and Methods

### 2.1. Cows, Diets, and Management

Eight midlactation Holstein dairy cows (176 ± 8 d in milk; 554 ± 13 kg body weight (BW), 3.12 ± 0.14 body condition score (BCS); mean ± SE) were randomly assigned to one of four treatments in a 4 × 4 replicated Latin square design with 4 21-day periods. Each period had 14 days of adaptation. Since dietary forage type and level were similar among diets, and treatments involved readily degradable grains, the 14-d was considered sufficiently optimal for rumen microbial adjustment to changes in GW level and particle size [19]. In addition, 14-d was set to avoid period by treatment interactions that are likely to occur with longer periods [20]. Treatments included 4 total mixed rations (TMRs) containing either (1) a higher (20%) or (2) a lower (10%) level of either (1) coarsely or (2) finely ground wheat grain (GW). Thus, GW level and grinding extent (GE) effects were determined in a 2 × 2 factorial arrangement. Ground wheat grain replaced ground barley grain when going from 10% to 20% GW (Table 1). Chopped alfalfa-hay-and wheat-straw-based TMRs with forage to concentrate ratio of 47.5 : 52.5 were fed to individual cows in equal portions 3 times daily at 0900, 1600, and 2300 h to permit about 5–10% daily orts, calculated based on the previous day's intake. Diets were formulated using NRC [13] for Holstein midlactation cows with 560 kg BW, 20 kg DMI, and 30 kg daily milk yield with 3.5% fat (Table 1). Cows were housed indoor in individual 3 × 4 m boxes equipped with concrete feed bunkers and metal water troughs. Cows were allowed 1 h of daily exercise 3 times daily prior to each milking. The experiment was conducted at the Dairy Facilities of the University of Zanjan Research Farm (Zanjan, Iran) according to the guidelines of the Iranian Council of Animal Care [21].

### 2.2. Grain and Forage Properties and Processing

Wheat (*Triticum spp.*) and barley (*Hordeum spp.*) grains were obtained from bulked sources representing grains fed in broad regions of Iran. Wheat grain DM contained 75% starch, 13% CP, 11% NDF, 3.2% ADF, and 2.3% ether extract. The respective values were 58%, 11%, 20%, 7%, and 2.2% for barley grain. Whole barley and wheat grains densities were 500 and 550 g/L, respectively. Rumen degradation rates of potentially degradable DM of corn, barley and wheat grains were 6%, 10%, and 12%/h, respectively. To produce the coarser and finer particle size treatments, WG was ground by passing through larger and smaller mesh size screens within a commercial hammer mill (model 5543 GEN, Isfahan Dasht, Isfahan, Iran), widely used by dairy farmers in Iran [8, 22]. Ground WG particle size distributions were determined using an electrical laboratory particle size separator-shaker with 7 round screens of decreasing mesh size on a bottom pan. The percentages of particles retained on the bottom pan and 0.8-mm, 1.2-mm, 1.6-mm, 3.0-mm, 5.0 mm and 8-mm screens were, respectively, 6.8%, 26%, 38%, 9.6%, 11%, 4.7%, and 3.3% for the coarser GW and 12%, 6.4%, 22%, 21%, 27%, 8.2%, and 3.2% for the finer GW. Alfalfa hay DM contained 13.1% CP and 41.6% NDF, and was chopped using an on-farm chopper machine (Agricultural Machinery Co., Tabriz, Iran) for an average theoretical chop length of 4 cm before mixing with concentrate. Forage and concentrate for individual cows were weighed daily and divided into 3 portions for 3 times daily feed delivery. At each feeding, forage and concentrate were mixed in the individual cow bunks.

### 2.3. DMI, Feed Analysis, and Total Tract Nutrient Digestibility

The amount of TMR offered and orts were measured daily from d 15–21 of each period to calculate DMI for individual cows. Samples of TMR were taken daily for individual cows during the 7 sampling d of each period. Feed and ort samples were pooled per cow per period and were oven-dried at 100°C for 24-h, ground to pass through 1-mm screen using a Wiley mill (Arthur H. Thomas Co., Philadelphia), and stored at −20°C until analyzed for nutrients. Feed samples were analyzed for CP (method 984.13; [23]), NDF (using heat-stable *α*-amylase and sodium sulfite), and ADF (method 973.18; [23]). Organic matter was determined by ashing the dried feed and feces for 8 h at 550°C (method 942.05; [23]). The acid insoluble ash (AIA; [22]) was used as an internal marker to determine total tract apparent DM and NDF digestibility. Particle size distribution of TMR samples was measured using Penn State Particle Separator (PSPS) [12]. For the PSPS analysis, the daily samples were composited to obtain 2 TMR and 2 forage samples per period. Physically effective NDF was estimated by multiplying dietary NDF% by (1) the proportion of DM retained on the 19.0-mm and 8.0-mm sieves of the PSPS (peNDF_>8_, [12]) and (2) the proportion of DM retained on the 19.0-mm, 8.0-mm, and 1.18-mm sieves of the PSPS (peNDF_>1.18_; [11]). After sieving, materials from each sieve were removed and dried at 55°C to determine DM content.

### 2.4. Milk Production and Composition

Cows were milked 3 times daily at 0800, 1500, and 2200 h in a milking parlor. Milk weights for individual cows were recorded daily during the entire experiment. Before milking, cows were monitored for udder inflammation and presence of milk clots to ensure that they were not infected by mastitis. Milk samples were collected during 2 consecutive days from 6 milkings into plastic vials containing potassium dichromate. Samples in plastic vials were analyzed separately for protein, fat, solid nonfats, and lactose using Milk-O-Scan (134 BN Foss Electric, Hillerod, Denmark). Milk components yield and content were calculated as a weighted mean for only the days that milk was sampled.

### 2.5. Blood Sampling and Analysis

Blood samples from individual cows were taken from tail veins at 0 and 2 h relative to feeding on the last day of each period. Blood was collected into anticoagulant-containing tubes and centrifuged at 1000 g for 15 min to harvest plasma for storage at −20°C until analyzed for *β*-hydroxybutyric acid (BHBA) (Ultraviolet method; Randox Kits, Cat. # RB1007 Antrim, UK), albumin (Bromocresol Green method), globulin (CHOD-PAR enzymatic method), and glucose (GOD-PAR enzymatic method) concentrations using commercial kits (Pars Azmun Laboratory, Tehran, Iran). The absorbance was read using spectrophotometer (Perkin- Elmer, Coleman Instruments Division, Oak Brook, IL, USA).

### 2.6. Fecal and Urine Parameters

For pH analysis, urine and fecal samples were collected twice daily at 1100 and 1800 h for the last 4 days of each period. Urine was collected by manual stimulation of the lower vulva. Urine samples in plastic vials were stirred and analyzed for pH immediately after sampling using a portable pH meter (HI 8314 membrane pH meter, Hanna Instruments, Villafranca, Italy). Daily fecal samples were taken from rectum at 1100 h and 1800 h for the entire 7-d sampling and stored at −20°C for digestibility measurements [22]. To determine fecal pH, a portion of it was mixed with an equal portion of distilled water and stirred for sufficient uniformity and thus for a representative pH value.

### 2.7. BW, BCS, and Back Fat Thickness (BFT)

Cow BCS was assessed by skilled individuals at the commencement of the first period and the end of all periods. A 5-point scoring scale, with 1 being an emaciated or extremely thin cow, and 5 being an obese or extremely fat cow, was used [24]. Ultrasound examination of BFT was made on the last d of each period to quantify subcutaneous fat deposition in the back area, as affected by treatments.

### 2.8. Statistical Analysis

Data were analyzed using MIXED procedures of SAS program [25]. The REML method was used to estimate least squares means, and Kenward-Roger method was used to calculate denominator degrees of freedom. Treatments effects on production variables were subjected to following linear model:

(1) $$\begin{array}{cc} {\text{Y}}_{cijps} & =\mu +{\text{GL}}_{i}+{\text{GE}}_{j}+{\left(\text{GL}\times \text{GE}\right)}_{ij}+{\text{PD}}_{p}+{\text{Sq}}_{s}\\ & \;+\text{Cow}{\left(\text{Sq}\right)}_{c(s)}+{\text{e}}_{cijps}, \end{array}$$

where, Y is response variable, GL is ground wheat grain level, GE is grinding extent, PD is period, Sq is square, and e is residuals. The “cow within square” was the main error term for testing the fixed square effect. For blood metabolites, the following linear model was computed:

(2) $$\begin{array}{cc} & {\text{Y}}_{cijpst}\\ & \;=\mu +\text{G}{\text{L}}_{i}+\text{G}{\text{E}}_{j}+{\left(\text{GL}\times \text{GE}\right)}_{ij}+{\text{Time}}_{t}\\ & \;\;+{\left(\text{GL}\times \text{Time}\right)}_{it}+{\left(\text{GE}\times \text{Time}\right)}_{jt}+{\left(\text{GL}\times \text{GE}\times \text{Time}\right)}_{ijt}\\ & \;\;+{\text{PD}}_{p}+{\text{Sq}}_{s}+\text{Cow}{\left(\text{Sq}\right)}_{c(s)}+{\text{e}}_{cijpst} \end{array}$$

where, Y is response variable, GL is ground wheat grain level, GE is grinding extent, PD is period, Sq is square, Time is blood sampling time, and e = residuals. Normality of distribution and homogeneity of variance for residuals were tested and ensured using PROC UNIVARIATE [25]. The *P*-values <0.05 were declared as significant, and *P* values ≤0.10 were declared as trends for significance. The standard errors of differences presented are those for pair-wise comparisons within the GL × GE interactions.

## 3. Results

### 3.1. Feed Intake and Milk Properties

Ground wheat grain (GW) inclusion rate affected DMI (*P* < 0.01) and tended to affect NE_L_ intake (*P* = 0.07), so that cows fed 10% GW had greater DMI and a tendency for greater NE_L_ intake than cows fed 20% GW (Table 2, *P* < 0.01). However, grinding extent (GE) and its interaction with GW inclusion rate did not affect DMI. Treatments did not affect milk production and composition including milk solids yield, milk energy density and yield, and milk fat to protein ratio (Table 2).

### 3.2. BW, BCS, and Back Fat Thickness (BFT)

Treatments did not affect BW and BCS changes (Table 3, *P* > 0.10). The BCS changes were not statistically different from zero (*P* > 0.10). However, an interaction of GW level and GE existed on BCS, so that GW at 10% instead of 20% of diet DM increased BCS when GW particles were coarser and decreased BCS when GW particles were finer. A tendency for a similar interaction (*P* < 0.10) of GW inclusion rate and GE was found on BFT examined by ultrasound (Table 3).

### 3.3. Total Tract Apparent Nutrient Digestibility and Fecal and Urine pH

Cows fed coarser versus finer GW tended to have greater total tract NDF digestibility (*P* = 0.09), as did cows on 10% versus 20% dietary GW (*P* = 0.07; Table 4). Total tract DM digestibility was similar among treatments. Whilst urine pH was unaffected by treatments, fecal pH tended to be higher at 20% versus 10% GW (*P* = 0.10, Table 4).

### 3.4. Blood Metabolites

Blood concentrations of albumin (*P* = 0.04) and total protein (*P* = 0.03) were greater in cows fed 10% GW than in cows fed 20% GW (Table 4). Blood BHBA concentrations increased with increasing dietary GW from 10% to 20% of DM (*P* = 0.01). Neither GW level, nor GE, nor their interaction affected other blood metabolites (Table 4).

## 4. Discussion

The present study provides novel practical in vivo insights into feeding lactating cows higher and lower dietary levels of finely and coarsely ground wheat grains (GW) in mixed rations with chopped alfalfa hay, wheat straw, and corn grain. Ground wheat has long been overlooked in dairy cattle nutrition [13, 26]. For its rapid degradation, GW has traditionally and not most reasonably been considered a risk to feed intake, rumen fermentation, and milk production. Another main reason for limited GW feeding to dairy cows is the GW popular consumption by humans and non-ruminant industries [1, 13]. Rapidly fermentable starch and proteins could predispose cows to subacute rumen acidosis (SARA), proinflammatory responses, milk fat depression, and laminitis [4, 27]. However, recent studies [7, 18] suggest that major but controlled dietary inclusion rates of steam-rolled WG (e.g., 20% of diet DM) are feasible for midlactaion cows. In addition, to maintain milk fat and functional rumen and cow metabolism, no sodium bicarbonate was required at 20% dietary steam-rolled GW [18]. Ground WG has also been feasibly fed to periparturient cows to improve DMI and Ca and energy balances [8]. The current data demonstrate that GW, regardless of its particle size, can feasibly be included in alfalfa hay based midlactation dairy diets at up to 20% of diet DM without compromising nutrient intake and milk properties. It should be considered that ground wheat grain replaced ground barley grain when going from 10% to 20% wheat grain. The greater DMI and NE_L_ intake of cows on 10% GW than that of cows on 20% GW could suggest that a combination of ground wheat (10.5%), barley (9.7%), and corn (5.8%) grains was more palatable than a blend of ground wheat (19.1%) and corn (5.8%) grains. In addition, the combined ground wheat, barley, and corn grains may have affected satiety signals differently than the blended ground wheat and corn grains. Moreover, the slightly greater dietary starch for the higher versus lower GW diet (19.5% versus 18.1%) could have contributed to decreased DMI of the higher GW diet. Furthermore, possible differences in portal dynamics of glucose might also contribute to the DMI differences via hepatic signals effects on feed intake regulation, despite the similar peripheral glucose concentrations among treatments. Due to the relatively lower NE_L_ density of the 10% GW diet than that of the 20% GW diet (1.60 versus 1.62 Mcal/kg), cows on 10% GW showed only a tendency for increased NE_L_ intake, compared to cows on 20% GW. The increased NE_L_ intake was consistent with the higher blood albumin and total protein at 10% instead of 20% GW, suggesting greater hepatic substrate supply and use for protein synthesis at 10% GW. Liver synthesizes albumin and other proteins for peripheral nutrient transport and optimum immune functioning. Doepel et al. [7] found no effects on DMI of feeding 20% steam-rolled WG to dairy cows in barley silage-based rations. Feed intake and blood metabolite data should be interpreted in light of the midlactation stage and dietary NDF levels of the current study. Midlactation scenarios would be expected to be more resistant to SARA and immune challenges than early and peak lactation cows.

The present study found no interactive and independent effects of GW inclusion rate and GE on milk production and composition of lactating cows. This finding along with DMI data suggest that GW, at 10% of diet DM, did not reduce diet palatability, a traditional concern that has restricted GW feeding to ruminants [26, 28]. Diets with 20% and 10% GW provided 19% and 17.4% cereal starch, respectively. These amounts are within the recommended maximum ranges of 20–25% to minimize health issues. Cows on 20% GW had similar milk energy output as cows on 10% GW while tending to consume less energy. Ground WG replacing barley grain was recently shown to favor prepartal DMI in cows and heifers [8, 17]. Consistent with the current data, rolled WG at lower (17.5%) and medium (19.7%) levels, respectively, corresponding to 24% and 31% dietary starch maintained milk fat and solids production, when compared to slowly degradable potato peelings [29]. In that study, at the low dietary starch level, WG also improved milk true protein content. These data support the hypothesis that feeding GW at major-controlled dietary levels would not compromise rumen and cow metabolism.

Adequately high milk fat and solids percentages and normal milk fat to protein ratios suggest no compromise of rumen conditions and function [10]. Most recent data [16, 30] demonstrated that steam rolling was not superior in DMI and milk production to fine grinding of barley grain at up to 30% barley grain. The numerical decrease in fecal pH of cows fed 10% versus 20% GW might in part be due to increased DMI at 10% GW. This could have increased postrumen flow of partially digested DM, thereby tending to increase hindgut fermentation and reduce fecal pH. Similar urine pH suggests no or little impact of treatments on extracellular fluids anion-cation balance and acidity [22]. The finding that GE did not affect DMI and milk production suggests that at up to 20% GW, its GE has little impact on cow performance. The midlactation stage and dietary NDF levels of the current study require important considerations because cows in midlactation would normally be less sensitive to rumen abnormalities and immune challenges than early lactation cows.

Since the cows were in midlactation and in positive energy balance, the tendency for increased blood BHBA by increasing GW from 10% to 20% has most likely an alimentary origin [31]. Increased peripheral blood BHBA concentrations could indicate a more extensive gastrointestinal epithelial metabolism of organic acids and output of BHBA [32]. Accordingly, gut epithelial BHBA supply may have tended to increase likely due to an increase in rumen degradation of cows fed 20% versus 10% GW. Total tract NDF digestibility was greater at 10% versus 20% GW when wheat particles were coarser, suggesting more favorable rumen conditions for fiber digestion in cows fed a blend of ground barley and wheat versus only 20% GW. The similar total tract DM digestibility among treatments was not unexpected, since differences in rumen digestibility of nonfiber diet components could disappear by the more extensive postrumen digestion processes including hindgut fermentation. Altogether, findings question any significant effects of feeding level and grinding extent of wheat grain on productivity of midlactation dairy cows fed an alfalfa-hay based diet.

## 5. Conclusions

Dietary inclusion rate (10% versus 20%) and grinding extent (coarser versus finer) of wheat grain and their interactions in chopped alfalfa-hay and wheat-straw-based mixed rations did not affect milk production and composition of midlactation cows. Ground wheat at 10% versus 20% GW increased DMI, and blood concentrations of total protein and albumin, while decreasing blood BHBA concentrations. Coarsely, and not finely, GW at 10% versus 20% of diet DM improved total tract apparent NDF digestibility. Data suggest that feeding midlactation cows finely and coarsely GW at 10% and 20% of diet DM with chopped alfalfa hay and ground corn is feasible. It should be considered that GW replaced ground barley grain when dietary GW was increased from 10% to 20%. Results imply the adequacy of the inexpensive grinding as an effective technique to process WG. These conclusions are made for midlactation cows on chopped alfalfa hay (42.8%) and wheat-straw-(4.8%) based mixed rations.

## Figures and Tables

**Table 1:** Dry matter based dietary ingredients at higher and lower inclusion rates of ground wheat grain in midlactation diets.

Ingredients and nutrients (DM based)	Dietary ground wheat grain	
	Lower (10%)	Higher (20%)
Alfalfa hay	42.8	42.8
Wheat straw	4.8	4.8
Ground barley grain	10.5	1.1
Ground wheat grain	9.7	19.1
Ground corn grain	5.8	5.8
Soybean meal	12.0	12.0
Whole cottonseed	6.8	6.8
Fish meal	3.6	3.6
Fatty acids-calcium soaps (powder)^1^	0.8	0.8
NaCl	0.42	0.42
Calcium carbonate	1.05	1.05
Sodium bicarbonate	1.05	1.05
Minerals and vitamins supplement^2^	0.63	0.63
DM %	92.5	93.5
CP %	18.5	18.6
Starch %	18.1	19.5
NDF %	39.5	40.5
peNDF_>8_^3^	18.4	18.0
peNDF_>1.18_^3^	37.3	34.8
NE_L_^4^, Mcal/kg	1.60	1.62
Calcium %	1.0	1.0
Phosphorous %	0.5	0.5

^1^Berg + Schmidt (GmbH & Co.) KG, Hamburg, Germany.

^2^Contained 250000 IU/kg vitamin A, 50000 IU/kg vitamin D, 1500 IU/kg vitamin E, 2.25 g/kg manganese, 120 g/kg calcium, 7.7 g/kg zinc, 20 g/kg phosphorus, 20.5 g/kg magnesium, 186 g/kg sodium, 1.25 g/kg iron, 3 g/kg sulfur, 1.25 g/kg copper, 14 mg/kg cobalt, 56 mg/kg iodine, and 10mg/kg selenium.

^3^NDF content of TMR or forage multiplied by their physical effective factor (pef) or the proportion of particles retained on the 19-mm, 8-mm, and 1.18-mm sieves of the PSPS [10, 11]. NDF content of TMR or forage multiplied by its pef or the proportion of particles retained on the 19-mm and 8-mm sieves of the PSPS [12].

^4^Estimated from NRC [13].

**Table 2:** Treatment effects on DMI and milk parameters of cows fed either finely or coarsely ground wheat grain (GW) at higher and lower inclusion rates.

	Grinding extent (GE)							
GW level (GL) %	Coarser		Finer			*P* value		
	10%	20%	10%	20%	SE	GE	GL	GE × GL
DMI, kg/d	19.7	19.4	20.1	19.4	0.22	0.22	<0.01	0.25
NE_L_ intake, Mcal/d	31.6	31.4	32.2	31.4	0.35	0.22	0.07	0.26
Milk yield, kg/d	25.4	25.1	25.1	25.5	0.44	0.83	0.99	0.28
Milk energy density^1^, Mcal/kg	0.700	0.695	0.696	0.699	0.01	0.97	0.88	0.58
Milk energy yield^1^, Mcal/d	17.69	17.30	17.50	17.68	0.42	0.75	0.72	0.34
Milk energy yield: DMI	0.90	0.90	0.87	0.92	0.02	0.72	0.20	0.12
Milk fat, %	3.42	3.37	3.52	3.39	0.11	0.39	0.26	0.60
Fat yield, kg/d	0.86	0.83	0.87	0.86	0.03	0.37	0.20	0.78
Milk protein, %	3.07	3.06	3.16	3.07	0.08	0.43	0.37	0.45
Protein yield, kg/d	0.78	0.77	0.79	0.78	0.02	0.35	0.41	0.98
Milk lactose, %	5.54	5.56	5.51	5.58	0.08	0.87	0.50	0.63
Lactose yield, kg/d	1.40	1.39	1.39	1.42	0.04	0.80	0.81	0.50
Milk SNF, %	9.77	9.71	9.66	9.75	0.10	0.56	0.83	0.30
SNF yield, kg/d	2.48	2.43	2.44	2.48	0.05	0.89	0.90	0.25
Total solids, %	13.39	13.02	13.27	13.33	0.24	0.57	0.36	0.21
TS yield, kg/d	3.39	3.24	3.34	3.37	0.09	0.47	0.38	0.14
Milk fat %: protein %	1.11	1.10	1.11	1.11	0.02	0.81	0.41	0.77

^1^Calculated based on milk components [13]: NE_L_ (Mcal/kg) = (0.0929 × fat %) + (0.0547 × crude protein %) + (0.0395 × lactose %).

**Table 3:** Treatment effects on body weigh (BW), body condition score (BCS), and back-fat thickness in cows fed either finely or coarsely ground wheat grain (GW) at higher and lower inclusion rates.

	Grinding extent (GE)							
GW level (GL) %	Coarser		Finer			*P* value		
	10%	20%	10%	20%	SE	GE	GL	GE × GL
BW, kg	588.1	595.6	591.4	595.9	6.30	0.68	0.19	0.74
BW changes, kg/d	0.50	0.73	0.61	1.06	0.38	0.42	0.22	0.70
BCS	3.34	3.19	3.03	3.25	0.08	0.05	0.61	0.01
Unit BCS changes	0.12	0.00	−0.19	−0.01	0.13	0.11	0.75	0.12
Back fat thickness, mm	21.6	20.4	21.4	23.0	1.00	0.10	0.82	0.07

**Table 4:** Treatment effects on blood metabolites, fecal and urine pH, and total tract apparent DM and NDF digestibility in cows fed either finely or coarsely ground wheat grain (GW) at higher and lower inclusion rates.

	Grinding extent (GE)							
GW level (GL) %	Coarser		Finer			*P* value		
	10%	20%	10%	20%	SE	GE	GL	GE × GL
NDF digestibility, %	34.8	32.0	32.1	31.9	1.10	0.09	0.07	0.11
DM digestibility, %	62.1	60.7	61.0	60.4	1.04	0.34	0.27	0.56
Glucose, mg/dL	51.1	51.6	50.8	51.1	1.70	0.84	0.81	0.96
Total protein, g/dL	8.5	8.2	8.6	8.4	0.08	0.38	0.03	0.42
Albumin, g/dL	3.84	3.75	3.83	3.68	0.07	0.42	0.04	0.56
Globulin, g/dL	4.62	4.46	4.64	4.67	0.14	0.23	0.50	0.32
BHBA, mol/L	0.53	0.64	0.55	0.64	0.07	0.74	0.01	0.71
Fecal pH	6.73	6.77	6.71	6.77	0.03	0.74	0.10	0.66
Urine pH	8.11	8.08	8.09	8.03	0.04	0.45	0.33	0.70
